# Supplementary material for: Transparent comparisons of Emergency-Department prioritization policies: integrating tail risk, target attainment, and utility analysis
Source: PLoS One. 2025 Dec 31;20(12):e0326722. doi: 10.1371/journal.pone.0326722 (PMC12755832; doi:10.1371/journal.pone.0326722)
Supplement: S1 File — (DOCX) [file pone.0326722.s001.docx]

**Appendix A: Patient Prioritization Strategies**

Throughout this manuscript, we illustrate our evaluation framework using nine distinct prioritization strategies. Three of these are well-established in the existing literature, three are novel strategies developed for this study, and the remaining three are composite strategies that combine elements of the novel approaches to capture their complementary strengths.

All strategies are applied exclusively to patients with Emergency Severity Index (ESI) levels 2 through 5. ESI 1 patients, representing the most critical cases, always receive immediate attention and are not subject to prioritization rules. As expected, our simulations confirm that ESI 1 patients exhibit similar key performance indicators (KPIs) across all strategies. Accordingly, we do not report results for ESI 1 patients in the analysis.

In our simulation model, each strategy is implemented at the point when a patient enters the physician queue. While the same logic could be extended to other service points such as nurse or radiology queues, we focus on the physician and bed queues, treating other stages as congestion-dependent time delays (see Appendix C for details). We do not assume that any single strategy is optimal across all queue types; for instance, a strategy effective for physician queues may be ill-suited for nurse queues. To avoid confounding effects, we restrict our evaluation to a single queue context per strategy.

The first strategy is **First-Come-First-Served (FCFS)**, in which patients are selected strictly by the order of their arrival to the queue, independent of acuity level or length of stay (LOS).

The second strategy, **Acuity-Based FCFS**, prioritizes patients with higher acuity. Ties within an acuity level are resolved by FCFS.

The third strategy is the **Accumulating Priority Queue (APQ)**, which integrates both acuity and LOS. Each patient’s LOS is multiplied by a weight based on their acuity level, and the patient with the highest resulting value is selected. In this study, all strategies that require acuity weights use values of 5, 3, 1, and 1 for ESI levels 2 through 5, respectively. Mathematically, the priority score $PS$ is defined as $PS=W_{a}t$, where $W_{a}$ is the weight for acuity level $a$ and $t$ is the current LOS in minutes.

We next developed a set of novel patient prioritization strategies guided by four design objectives: (i) ease of implementation for ED stakeholders, (ii) no additional workload for ED staff, (iii) transparency and interpretability of prioritization decisions, and (iv) strategic targeting of known bottlenecks in ED operations.

Our first novel strategy was formulated as a variant of the standard APQ by performing transformations using the weight $W_{a}$ and current LOS $t$. To explore alternative formulations, we evaluated several variations that modify the interaction between time and acuity level. Although not included in the final analysis, the following variants were considered:

**Exponential Accumulating Priority Queue (EAPQ):**

$$PS=W_{a}(e^{\frac{t}{T}}-1),$$

where $T>0$ is a scaling parameter. For $t\ll T$, the score approximates $PS\approx W_{a}t/T$, recovering the linear structure of APQ.

**Polynomial Accumulating Priority Queue (PAPQ):**

$$PS=W_{a}*(1+t^{\alpha} ),$$

with $\alpha\geq1$ as a shape parameter. The curvature of this score increases with $\alpha$, introducing non-linear acceleration in priority accumulation.

**Threshold Quadratic Accumulating Priority Queue (TQAPQ):**

$$PS=\left\{ \begin{aligned} W_{a}t , t<T \\ W_{a}t+{\lambda\left( t-T \right)}^{2}, t\geq T \end{aligned} \right.,$$

Where $T>0$ and $0\leq\lambda\leq1$ control the onset and intensity of quadratic escalation.

The variant selected for inclusion in this study is the **Additive Accumulating Priority Queue (AAPQ)**, defined by

$$PS=W_{a}+ \beta t,$$

where $\beta\in[0,1]$modulates the contribution of LOS. We conducted a sensitivity analysis and selected $\beta=0.001$, chosen to reflect the desired magnitude of influence without requiring fine-tuning. The priority score is largely insensitive to small changes in $\beta$ (e.g., 0.0005 or 0.005 yield similar rankings), as the acuity term $W_{a}$​ dominates. This stability across values makes AAPQ easy to implement and adaptable across EDs without site-specific calibration.

Moreover, for small $\beta$, AAPQ simplifies operationally to a variant of Acuity-Based FCFS: patients are primarily ordered by acuity level, with ties broken by current LOS rather than queue entry time. This logic is intuitive and requires minimal computation—one can identify the patient with the highest acuity and resolve ties by selecting the one who has waited longest. This satisfies our implementation criteria and retains alignment with clinical reasoning.

The next strategy we introduce is the **Low Workload Physician (LWP) Strategy**, designed to account for real-time physician workload when assigning patients. This approach preferentially routes patients to physicians with fewer active assignments, promoting a more balanced distribution of workload and reducing the risk of overburdening any single provider.

We classify LWP as an “add-on” strategy: rather than replacing an existing prioritization rule, it operates conditionally alongside a default strategy. Specifically, the default strategy governs patient selection under normal circumstances, but the LWP rule supersedes it when certain conditions are met. In our implementation, APQ serves as the default strategy.

The LWP strategy introduces a single parameter, $m$, which specifies the minimum number of active patients a physician must have before the default strategy is restored. A patient is considered *assigned* to a physician if they are under the physician’s care and not in the process of being discharged. A patient is considered in the *general queue* if they have been moved from the waiting room into a bed but have not yet been assigned to a physician.

The LWP rule is activated when the following conditions are satisfied:

1. Condition 1: There exists at least one physician with $\leq m$ assigned patients.
2. Condition 2: That physician has fewer assigned patients than any other physician.
3. Condition 3: There is at least one patient in the general queue.

If all three conditions hold, the patient in the general queue with the highest APQ score is assigned to the identified physician. If any condition is not satisfied, patient assignment proceeds under the default APQ strategy.

We also explored variants of Conditions 1–3, such as removing certain conditions or redefining physician workload using composite metrics that incorporate both the number and resource intensity of assigned patients. However, in the scenarios we evaluated, the original formulation of LWP yielded the most favorable results. That said, we emphasize that "best" is used contextually, as each strategy entails trade-offs depending on the operational objectives and ED configuration. Our sensitivity analysis for LWP was limited in scope, intended primarily to select a small set of promising strategies for evaluation. A more comprehensive sensitivity study across all proposed strategies and their respective parameters represents an important direction for future research.

Our third novel strategy is the **Partial Fast Track (PFT)** approach. Similar to LWP, PFT functions as an *add-on* strategy layered on top of a default prioritization rule; in our implementation, the default strategy is again set to APQ.

PFT designates one physician to prioritize low-acuity patients, while all remaining physicians continue to select patients according to the standard APQ rule across all acuity levels. Importantly, this does not exclude the other physicians from treating low-acuity patients—they continue to do so within the standard prioritization process. Rather, the purpose of PFT is to introduce a targeted prioritization mechanism to expedite care for lower-acuity patients.

When the designated low-acuity physician selects a patient from the general queue (i.e., patients who have been placed in a bed but not yet assigned a physician), the following logic is applied:

1. If any **ESI 5** patients are present, the physician selects the one with the earliest arrival time.
2. If no ESI 5 patients are available but **ESI 4** patients are present, the physician selects the ESI 4 patient with the earliest arrival time.
3. If neither ESI 5 nor ESI 4 patients are available, the physician defaults to the standard APQ rule applied across all remaining patients.

As with all strategies in this study, ESI 1 patients are excluded from the prioritization logic and always receive immediate attention, regardless of physician designation or prioritization strategy.

While the low-acuity physician prioritizes ESI 4 and ESI 5 patients when they are present, they are still permitted to treat higher-acuity patients if no low-acuity patients remain in the queue. Likewise, the other physicians continue to treat patients across all acuity levels, ensuring flexibility and full utilization of available provider capacity.

Finally, we developed **combination strategies** to assess whether integrating multiple prioritization mechanisms could yield improved performance relative to using any individual strategy alone. Specifically, we examined combinations of the AAPQ, LWP, and PFT strategies.

Since both LWP and PFT are formulated as *add-on* strategies, they can be layered on top of any base rule. Accordingly, we replaced the default APQ rule with AAPQ in both cases, resulting in the combination strategies **AAPQ-LWP** and **AAPQ-PFT**, respectively.

We also constructed a three-way combination, **AAPQ-LWP-PFT**, which sequentially applies all three strategies. In this configuration, PFT conditions are evaluated first; if none are met, the LWP logic is applied; and if the LWP conditions also fail, the strategy defaults to AAPQ.

In summary, the novel and combination strategies we developed—AAPQ, LWP, PFT, and their composites—were specifically designed to be both operationally practical and responsive to known inefficiencies in emergency department patient flow. As demonstrated in our illustrative analysis (Table 5 of the main text), these strategies consistently performed well, particularly in improving throughput and balancing workload without adding complexity to implementation. Their simplicity, interpretability, and modular design make them promising candidates for future exploration. Accordingly, a natural extension of this work is to conduct broader sensitivity analyses and real-world validation studies to further investigate the generalizability and performance of these and similar strategies across diverse ED settings.

**Appendix B: Utility Functions**

**B.1 ED Utility Function Literature Review**

Evaluation of patient-prioritization policies in a discrete-event simulation requires a scalar performance measure that respects managers’ stated goals for each patient cohort. When those goals act as reference points and shortfalls are viewed as more harmful than equivalent surpluses are beneficial, the utility (or loss) function must be asymmetric. Empirical research in psychology and behavioral economics shows that decision makers typically overweight losses relative to gains at a ratio of roughly two-to-one [1, 2]. Consistent with loss-aversion theory, interviews with 617 emergency-department clinicians and managers across Australia, New Zealand, Canada, and the UK revealed that stakeholders experienced markedly greater disutility when length-of-stay targets were missed than the modest satisfaction reported when those targets were slightly exceeded [3]. Three broad families of utility functions have emerged in the last two decades for modelling such preferences in health-care and operations-research applications.

The piecewise-linear weighted-deviation model—often labelled weighted goal programming—remains the most common choice in applied simulation research. For each performance dimension $i$ it defines separate non-negative variables for undershoot, $n_{i}=max\{0,g_{i}-x_{i}\}$ and overshoot, $p_{i}=max\{0,x_{i}-g_{i}\}$*,* and aggregates them through a linear combination $\sum_{i} w_{i}^{-}n_{i}+w_{i}^{+}p_{i}$. Studies across manufacturing [4], transport planning [5] and, more recently, emergency-department flow analysis [6] show that large ratios $w_{i}^{-}/w_{i}^{+}$ faithfully reproduce managerial priorities while preserving linear-programming tractability. Since slopes are constant on each side of the goal, marginal penalties remain transparent to non-technical stakeholders and sensitivity analysis on the weight ratio is straightforward.

Although piecewise linearity is intuitive, the derivative discontinuity at the goal can hinder gradient-based optimization or learning algorithms. To smooth the kink while retaining loss aversion, several authors have imported prospect-theoretic value functions into operations research. Abdellaoui et al. (2007) estimate an S-shaped function that is convex for $x<g$ and concave for $x>g$, with a multiplicative loss-aversion coefficient $\lambda>1$ [1]. A smooth, asymmetric alternative is the linear–exponential (LINEX) loss, defined as $U\left( d \right)=\exp\left( \alpha d \right)-\alpha d-1$ where the sign and magnitude of $\alpha$ determine whether undershoot or overshoot is more heavily penalized [7]. In particular, Azimaee *et al.* applied a LINEX specification within a Bayesian multilevel framework for surgical force estimation, exploiting its exponential penalty on negative deviations to reflect steep disutility when models underestimate critical tissue forces [8]. Both the S-shaped prospect value function and the LINEX loss reduce to a weighted-deviation (piecewise-linear) form for small values of $|d|$, yet they remain continuously differentiable at the reference point, thereby facilitating adjoint-based calibration in simulation and optimization routines.

When policy makers insist that no dimension may fall below its target, research advocates a maximin or Chebyshev formulation that evaluates performance by the largest normalized shortfall. Flavell’s (1976) Chebyshev goal-programming variant [9], and its stochastic extensions developed over the past decade for sustainable health-service design [10], effectively impose infinite loss aversion on the worst-performing metric. Recent machine-learning work has proposed differentiable approximations, such as the split-function exponential–logarithmic (SFELLA) transform, which temper numerical instability while still forcing balanced improvements [11]. These “soft maximin” utilities are particularly attractive for real-time control where a reinforcement-learning agent must learn gradients from simulation noise.

Under stochastic arrivals and service times, all three functional families embed naturally in expected-utility objectives, but their implications for risk differ. Piecewise-linear loss combined with uncertainty places primary weight on the expected magnitude of shortfall, whereas Chebyshev-type utility implicitly minimizes the probability of any shortfall, linking it to chance-constrained programming and the safety-first principle [12]. Downside-focused risk measures can be linked directly to specific utility‐function classes. In particular, lower‐partial‐moment (LPM) measures capture the expected shortfall below a target and, when taken to second order, impose a quadratic penalty on negative deviations [13]. By contrast, exponential‐loss utilities converge to conditional value-at-risk (CVaR) minimization as the aversion parameter grows: Rockafellar and Uryasev (2000) demonstrate that, in the limit of large loss aversion, minimizing an exponential penalty on shortfalls is equivalent to minimizing CVaR, thereby embedding coherent tail‐risk control within the utility framework [14]. Consequently, analysts should choose among LPM‐based (quadratic on shortfalls), exponential‐loss (tail‐sensitive), or CVaR‐equivalent formulations to match managerial tolerance for occasional breaches.

In summary, contemporary literature suggests that piecewise-linear weighted deviations are the most interpretable and remain computationally convenient for deterministic discrete-event simulation, smooth prospect-theoretic or LINEX utilities are preferred when gradient information is required or when very long waits incur super-linear disutility, and Chebyshev or soft-maximin functions are appropriate when any goal failure is unacceptable. The empirical loss-aversion ratios reported in health-care studies justify weighting under-performance at least twice as strongly as over-performance. Adopting these guidelines aligns the evaluation of patient-prioritization strategies with observed stakeholder preferences and with best practice across multi-objective optimization, behavioral economics, and recent emergency-department analytics.

**B.2 Elliptical Utility Function**

The elliptical utility described below (denoted $U_{1}$​ in the main text) is one of the functions we used to evaluate patient-prioritization strategies in our numerical experiments. It satisfies the qualitative requirements identified in Section B.1—penalizing shortfalls more than overshoots, allowing differential weights across acuity cohorts, and remaining continuously differentiable—yet it is **only one plausible specification**. We make no claim that $U_{1}$​ perfectly reflects every stakeholder’s preferences.

**B.2.1 Definition**

Let

$$x=\left( \begin{matrix} x_{L} \\ x_{M} \end{matrix} \right), g=\left( \begin{matrix} x_{L} \\ x_{M} \end{matrix} \right)\in(0, 100]^{2},$$

denote, respectively, the realized and aspirational discharge percentages for the low- and mid-acuity cohorts.
Define the diagonal weight matrix

$$W\left( \lambda\right)=\left( \begin{matrix} 1 & 0 \\ 0 & \lambda\end{matrix} \right), \lambda\geq1,$$

where a single parameter $\lambda$ captures the relative importance of the mid-acuity dimension.

Introduce the element-wise positive and negative part operators

$$s\left( x \right)=[g-x]_{+}, o\left( x \right)=[x-g]_{+}, [z]_{+}:=max\{0, z\},$$

so that $s_{i}(x)$ records short-fall, and $o_{i}(x)$ records overshoot in coordinate $i$.
The scalar objective is formulated as

$$U_{\lambda,\delta}=-s(x)^{\top}W\left( \lambda\right)s\left( x \right)+\delta o(x)^{\top}W\left( \lambda\right)o\left( x \right), 0<\delta<1,$$

This equation can be written explicitly as

$$U_{\lambda,\delta}=-([g_{L}-x_{L}]_{+}^{2}+\lambda[g_{M}-x_{M}]_{+}^{2})+\delta([x_{L}-g_{L}]_{+}^{2}+\lambda[x_{M}-g_{M}]_{+}^{2}).$$

If overshooting the goal should not be rewarded, then setting $\delta$ close to zero is appropriate; setting it exactly to zero is weakly dominated and may result in inferior behavior under certain conditions.

Table B.1 Explanation of the Components of the Elliptical Utility Function

| **Symbol** | **Meaning** |
| --- | --- |
| $[g_{L}-x_{L}]_{+}^{2}$​ | Squared low-acuity short-fall. |
| ${\lambda[g}_{M}-x_{M}]_{+}^{2}$ | Squared mid-acuity short-fall amplified by relative-importance factor $\lambda$. |
| $\delta[x_{L}-g_{L}]_{+}^{2}$​ | Squared low-acuity overshoot, credited at the discount rate $\delta$. |
| $\delta\lambda[x_{M}-g_{M}]_{+}^{2}$ | Squared mid-acuity overshoot, likewise discounted and up-weighted by $\lambda$. |

**B.2.2 Mathematical properties**

The gradient of $U_{\lambda,\delta}$​ is positive in any dimension that still fails to meet its goal, ensuring that incremental improvements always raise utility until the target is reached. At the goal $x_{i}=g_{i}$​ the slope drops from $2w_{i}(x_{i}-g_{i})$, to $2\delta w_{i}(x_{i}-g_{i})$; because $0<\delta<1$, marginal gains are attenuated but remain non-negative once performance moves into the overshoot region. The level sets $U_{\lambda,\delta}=c$ coincides with the interior arcs of ellipses centered at $g$. Inside the “goal box’’ $(x_{L}\leq g_{L}, x_{M}\leq g_{M})$ each contour is a true ellipse whose aspect ratio equals $\sqrt{\lambda} :1$; outside the box, the contour flattens in any direction where the corresponding coordinate has already cleared its goal, reflecting the plateau of discounted rewards. Because the utility is a strictly monotone transform of a weighted squared distance for all points that have at least one unmet goal, it induces a total ordering on simulation outputs; generic ties arise only when two policies coincide on every goal-relevant coordinate.

**B.2.3 Data-driven calibration of** $\boldsymbol{\lambda}$ **and** $\boldsymbol{\delta}$

The relative-importance weight $\lambda$ captures how much additional disutility managers attach to a one-unit shortfall in the mid-acuity cohort compared with the same shortfall in the low-acuity cohort. One empirical procedure presents decision makers with two historical or simulated schedules deemed “equally acceptable’’ and solves

$$[g_{L}-x_{L}^{(1)}]_{+}^{2}+\lambda[g_{M}-x_{M}^{(1)}]_{+}^{2}=[g_{L}-x_{L}^{(2)}]_{+}^{2}+\lambda[g_{M}-x_{M}^{(2)}]_{+}^{2},$$

yielding a point estimate $\hat{\lambda}$. Repeating this paired-comparison exercise across multiple schedule pairs produces a distribution of $\lambda$ values; maximum-likelihood or Bayesian hierarchical models can then summarise central tendency and uncertainty.

The overshoot-damping coefficient $\delta$ reflects how quickly marginal utility decays once a cohort’s goal is surpassed. Calibration may begin with stated-preference surveys in which stakeholders rate the desirability of incremental improvements beyond the target. Regressing these ratings on the squared overshoot term ${(x_{i}-g_{i})}^{2}$ recovers an empirical slope ratio, providing an estimate of $\delta$. Alternatively, revealed-preference methods can infer $\delta$ from historical scheduling choices: if managers systematically trade a large gain in one cohort for a small gain in the other, the observed trade-off implies a particular $\delta$ consistent with their willingness to accept diminishing returns.

**B.3 Goal-Attainment Utility Function**

The second utility function we employ, denoted $U_{2}$​ in the main text, measures performance by the *average* distance that still separates each cohort from its target. Unlike the elliptical utility $U_{2}$​, which continues to grant (discounted) credit once a goal is surpassed, $U_{2}$​ is indifferent to overshoot and concentrates all attention on whatever deficit remains. This “gap-closing’’ perspective aligns with managerial settings in which resources are re-deployed as soon as any cohort has met its service-level requirement. Despite multiple differences between these utility functions, our illustrative analysis finds consistent conclusions when using both utility specifications.

**B.3.1 Definition**
For the low- and mid-acuity cohorts let

$$s_{L}=[g_{L}-x_{L}]_{+}, s_{M}=[g_{M}-x_{M}]_{+}.$$

A single scaling parameter $0< \alpha\leq1$ down-weights the low-acuity gap relative to the mid-acuity gap.

The mean scaled shortfall is

$$\theta_{\alpha}\left( x \right)=\frac{1}{2}\left( \alpha s_{L}+s_{M} \right),$$

and the goal-attainment utility is defined as

$$U_{\alpha}\left( x \right)=1-\theta_{\alpha}(x).$$

**B.3.2 Analytic properties**

Level sets of constant utility satisfy $\alpha s_{L},+s_{M}=2(1-c)$; geometrically they are parallel lines of slope $-\alpha$ that translate toward the origin as average goal attainment improves. Neither dimension can dominate the other: even if the low-acuity gap closes entirely, any residual mid-acuity shortfall continues to depress utility, and vice-versa. Conversely, once a cohort’s performance exceeds its goal the corresponding shortfall becomes zero and further improvements on that axis leave $U_{\alpha}$ unchanged, ensuring that managerial focus shifts to the remaining deficit.

**B.3.3 Parameter calibration**

The weight $\alpha$ governs the relative importance of low-acuity versus mid-acuity delays. One operational approach elicits indifference curves from stakeholders: pairs of schedules are presented in which a reduction of $\Delta$ minutes in low-acuity LOS is traded against a reduction of $\Delta^{*}$ minutes in mid-acuity LOS. Setting $\alpha=\Delta^{*}/\Delta$ reproduces the stated equivalence between the two trade-offs. In the numerical illustrations we adopted $\alpha=0.6$, implying that a one-minute delay for a mid-acuity patient is valued as highly as a 1.67-minute delay for a low-acuity patient.

The remaining numerical inputs—$\lambda=1.5$ and $\delta=0.3$ for $U_{1}$​, along with cohort-specific LOS goals of $(50\%, 50\%)$, $(100\%, 90\%)$, and $(100\%, 100\%)$ at 3-, 5-, and 7-hour thresholds—were selected purely for illustration. In practice, all parameters should be calibrated jointly with local clinicians and administrators to reflect site-specific service priorities and risk tolerance.

Table B.2 Hyperparameters Used in the Illustrative Application

| **Hyperparameter** | **Value Used in Illustrative Application** |
| --- | --- |
| $\lambda$ | 1.5 |
| $\delta$ | 0.3 |
| $\alpha$ | 0.6 |
| LOS Goal at 3-hour Threshold | $\left( 50\%, 50\% \right)$ |
| LOS Goal at 5-hour Threshold | $\left( 100\%, 90\% \right)$ |
| LOS Goal at 7-hour Threshold | $\left( 100\%, 100\% \right)$ |

**B.4 Utility Metric Comparison**

Table B.3 Comparison of the Elliptical and Goal-Attainment Utilities

| **Criterion** | **Elliptical utility** $U_{\lambda,\delta}$**​** | **Goal-attainment utility** $U_{\alpha}$ |
| --- | --- | --- |
| Preference parameter | $\lambda\geq1$ (importance ratio) | $\alpha\in(0, 1]$ (low-acuity down-weighted by $\alpha$) |
| Overshoot treatment | Rewarded at reduced rate $\delta$ | No additional credit (truncated) |
| Scale dependence | Absolute deviations | Scale-free |
| Contour geometry | Ellipse truncated at targets | Parallel lines (slope = $-\alpha)$ |
| Typical use-case | Settings where surpassing benchmarks is encouraged | Settings where effort after reaching a target should redirect to lagging areas |

**B.5 Visualizing robustness across multiple ED configurations with covariance ovals**

All results in the main text are reported for one calibrated emergency-department configuration at a time. A natural next step is to compare how each prioritization strategy behaves simultaneously across several plausible configurations—for example, alternative demand levels, boarding policies, or staffing mixes. One compact way to summarize this joint performance is to plot the *covariance oval* (also called a covariance ellipse) of the low- and mid-acuity outcomes obtained when the strategy is re-evaluated under $N$ perturbed parameter settings.

**B.5.1 Construction of the oval**

Let $x^{(k)}=(x_{L}^{\left( k \right)},x_{M}^{(k)})$, $k=1, \ldots, N$, denote the pair of discharge-rate percentages delivered by a fixed strategy in each of the $N$ alternative ED scenarios. The empirical mean and covariance matrix are

$$\bar{x}=\frac{1}{N}\sum_{k=1}^{N} x^{(k)}, \Sigma=\frac{1}{N-1}\sum_{k=1}^{N} {(x}^{(k)}-\bar{x})(x^{(k)}-\bar{x})^{\top}.$$

For any pre-specified confidence mass $\gamma$ (e.g., $\gamma=0.68$, the analogue of one standard deviation in the univariate case) the oval is the locus

$$\left( x-\bar{x} \right)\Sigma^{-1}\left( x-\bar{x} \right)^{\top}= \chi_{2, \gamma}^{2},$$

with $\chi_{2, \gamma}^{2}$ the upper $\gamma$ quantile of the chi-square distribution with two degrees of freedom (e.g., $\chi_{2, 0.68}^{2}\approx2.28$). If the sampling distribution of $(x_{L},x_{M})$ were exactly bivariate normal, the ellipse would enclose approximately $\gamma$ of the probability mass; even when normality fails, it still provides a faithful second-moment summary.

**B.5.2 How to interpret shape and position**

The center $\bar{x}$ represents the expected performance averaged over the $N$ configurations. Its proximity to the clinical goal point immediately indicates whether the strategy is, *on average*, compliant with service targets. The geometry of the oval then reveals how sensitive that compliance is to configuration changes. A wide horizontal semi-axis (large $\sqrt{\Sigma_{LL}}$) signals substantial variability in low-acuity performance, whereas a tall vertical semi-axis (large $\sqrt{\Sigma_{MM}}$​) indicates volatility in the mid-acuity cohort. The tilt of the oval is governed by the sign of the off-diagonal entry $\Sigma_{LM}$​: a positive tilt shows that improvements (or deteriorations) in one cohort tend to be accompanied by similar movements in the other, whereas a negative tilt betrays a trade-off relation. Eccentricity, measured by the ratio of the square-roots of the eigenvalues of $\Sigma$, identifies whether the scatter is essentially one-dimensional; a highly elongated, slim oval implies that perturbations push performance mainly along a single composite direction, so stabilising that direction would simultaneously stabilise both outcome metrics. Finally, the area of the oval, proportional to
$\det\Sigma$, provides an overall gauge of robustness: smaller area means the strategy is less sensitive to the heterogeneity embodied in the $N$ scenarios.

Figure B.1 concretizes the abstract discussion of Section B.5 by displaying the one-standard-deviation ovals for three representative strategies at the three-hour LOS threshold. Several qualitative insights become transparent at a glance. First, the near-circular shape of the AAPQ-LWP-PFT oval implies that configuration changes perturb low- and mid-acuity performance by comparable amounts and in roughly the same directions, suggesting a robust, well-balanced policy. In contrast, the highly flattened, horizontally oriented FCFS oval indicates that the same perturbations cause large swings in the low-acuity cohort while leaving the mid-acuity cohort relatively unaffected; a hospital relying on FCFS should therefore expect volatile compliance with low-acuity targets whenever demand or staffing shifts. Finally, the moderate eccentricity and positive tilt of the AAPQ-LWP oval reveal a middle ground: variability is present but more evenly shared, so incremental improvements aimed at stabilizing one cohort are likely to benefit the other. Although no managerial conclusions should be drawn from a single threshold or the limited scenario sample shown, the figure illustrates how covariance ovals can expose differences in strategy robustness that remain hidden when each configuration is inspected in isolation.

A visual juxtaposition of the oval with the target point therefore conveys both *expected* goal attainment and the *reliability* of that attainment across multiple ED environments. Extending the evaluation criteria in our paper to integrate such multi-configuration summaries is a promising avenue for future research, allowing analysts to identify strategies that are not merely optimal for a single setting but remain effective—and predictable—across the spectrum of operating conditions a health system is likely to encounter.


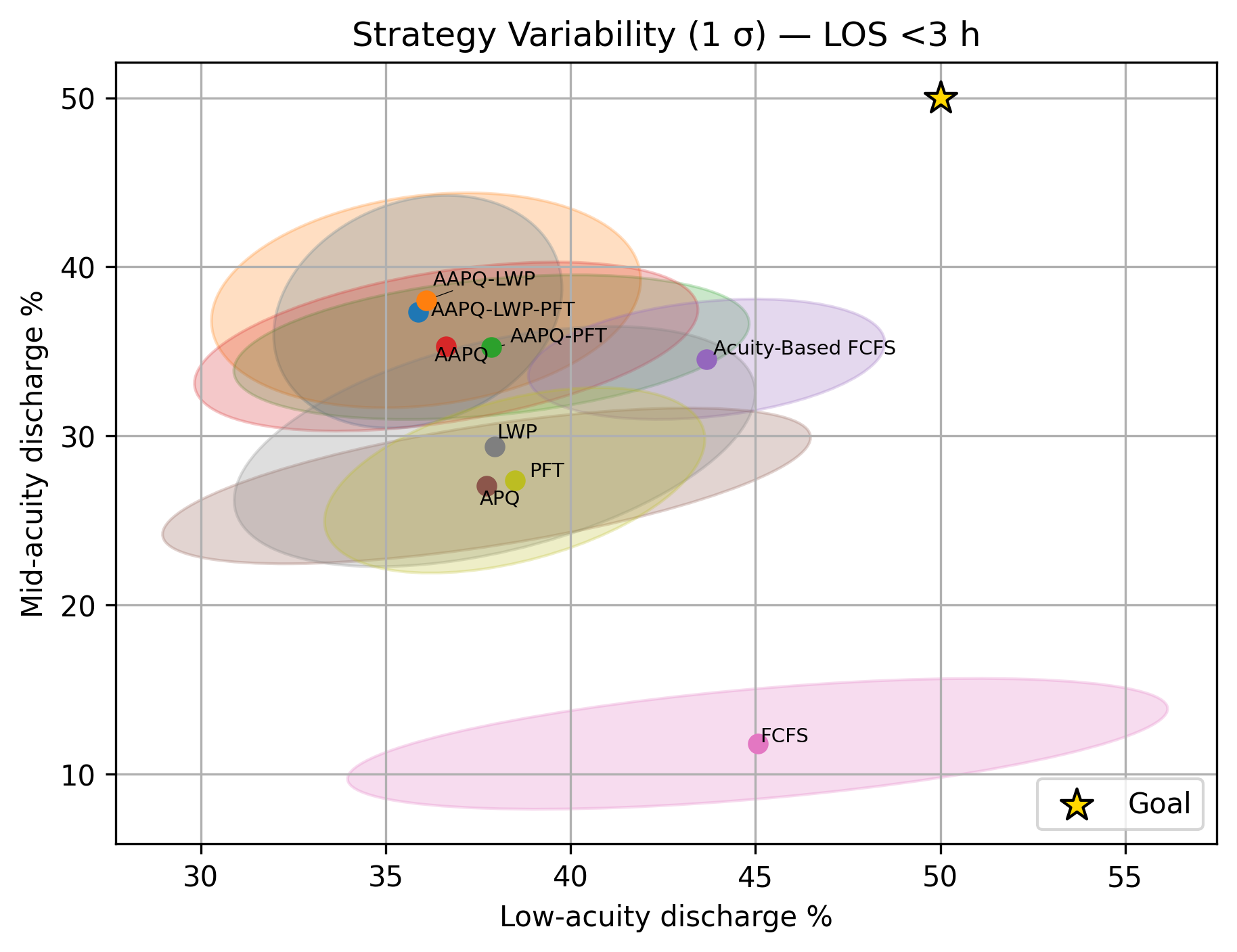


Fig. B.1 **Illustrative 1-σ Covariance Ovals at the 3-Hour LOS Threshold**. Each colored oval summarizes, for a single patient-prioritization strategy, the joint variability of low- and mid-acuity discharge percentages obtained when that strategy is reevaluated under ten perturbed emergency-department configurations. The star marks the target point (50%, 50%). Strategies exhibiting nearly circular ovals (e.g., AAPQ-LWP-PFT) display balanced, low-correlated variability, whereas strongly elongated ovals (e.g., FCFS) reveal pronounced sensitivity along one dimension—in this case the low-acuity axis. Intermediate behavior is observed for AAPQ-LWP, whose moderate eccentricity suggests coupled, but less extreme, trade-offs between cohorts. The figure is intended purely as a visual exemplar of the covariance-oval concept; the nine underlying scenarios are not discussed individually.

**Appendix C: Discrete Event Simulation Details**

**Simulation Overview**

The simulation represents patient flow in an ED using a discrete-event simulation (DES) approach. Time advances from one event to the next (e.g., arrivals, task completions), rather than in fixed increments, thereby capturing the dynamic interplay of patient arrivals, service tasks, and resource availability more efficiently.

The DES model described herein was developed solely to *illustrate* the proposed evaluation techniques presented in the main manuscript. It is not intended to reproduce the exact workflow, staffing structure, or patient mix of any emergency department. Instead, the model abstracts and simplifies common ED processes in a controlled setting to highlight how different prioritization strategies affect patient outcomes under varied demand conditions. As such, results should not be interpreted as predictive for any specific institution but rather as illustrative demonstrations of methodological applicability.

**1. Patient Generation and Arrival Process**

Patients are generated based on a stochastic process, often assumed to follow Poisson arrivals over multiple days. The daily cycle is divided into time blocks (e.g., three shifts), beginning at 8:00 PM, 4:00 AM, and 12:00 PM, respectively. The 8:00 PM block is associated with the lowest arrival rate, while the 12:00 PM block exhibits the highest arrival rate. These time blocks and corresponding rates were selected to align with patterns reported in Stenson et al. [15] and Furian et al [16]. Additionally, no distinction was made based on the day of the week, as Stenson et al. [15] and Chaou et al. [17] reported similar patterns across all days.

Patients are classified into acuity levels (e.g., 1 to 5), which influence both their arrival patterns, and the complexity of care required. Higher-acuity patients (level 1) have the lowest arrival rates, whereas lower-acuity patients (levels 4 and 5) have the highest arrival rates. Patient pathways are modeled based on the eleven process paths described in Lee and Lee [18]. Higher-acuity patients are assigned higher probabilities of following later process paths, as these involve more intensive treatments and often include admission to an inpatient unit.

**2. Task Sequence and ED Workflow**

Upon arrival, every patient undertakes a series of tasks that mimic the ED workflow. Task completion times were assigned times similar to those reported in Lee and Lee [18].

1. **Triage:** Each patient is initially triaged, during which a brief evaluation occurs, and the patient’s condition is assessed.
2. **Waiting Room:** If no beds are available, the patient waits in a waiting room until a bed becomes available.
3. **Obtain a Bed:** Once a bed becomes available, the patient obtains a bed and is entered into a general physician queue. Any physician may select a patient waiting in the general queue.
4. **Physician Consultation:** The patient meets with the physician to assess their condition and perform any immediate treatment, if required.
5. **Additional Testing and Procedures:** Some patients may require diagnostic tests (e.g., imaging or lab work) and possibly nursing interventions. We approximate these procedures as “testing tasks” with their own durations and resource constraints.
6. **Repeat Physician Consultation:** If results from any test necessitate further review, the patient may return to the physician queue.
7. **Boarding (if admitted):** Patients requiring inpatient admission may board in the ED if a regular inpatient bed is not yet available. This step can significantly extend a patient’s stay.
8. **Discharge:** Once all necessary care is complete, the patient is discharged. The bed previously occupied undergoes a brief cleanup time before being available for the next patient.

**3. Resource Constraints and Queues**

**Beds and Waiting Room:** The number of ED beds is finite. When no bed is available at triage completion, patients must wait in a “virtual” waiting room. As soon as a bed opens, a patient from the waiting room is admitted to that bed.

**Over Capacity:** Our simulation is based on an ER with 30 beds. If the waiting room has an additional 30 patients (for a total capacity of 60), then newly arriving patients are turned away. In the High-Demand Scenario the turn away rate was approximately 17%-22% across all strategies, which is consistent with the Yellow Zone Ambulance Diversion rate of 20.3% in the intervention group of a study from Johns Hopkins Bayview Medical Center [19].

**Physicians:** Physician staffing levels can vary by shift, reflecting standard ED scheduling practices. The day is divided into three 8-hour shifts beginning at 12:00 AM, 8:00 AM, and 4:00 PM, respectively. The 12:00 AM shift has the lowest number of physicians, whereas the 4:00 PM shift has the most physicians. When multiple patients are waiting, each physician may handle one patient at a time. Patients either enter a general queue (for any available physician) or remain in a physician-specific queue if they have an assigned physician who must see them again later.

**Other Services:** Diagnostic procedures (e.g., imaging, laboratory tests) are influenced by capacity constraints and congestion effects. To simulate real-world bottlenecks, a busyness factor (BF) is incorporated, where increased demand leads to longer test durations. The busyness factor is defined as $BF=1+ \frac{N}{B}$ where $N$ represents the number of patients currently requiring the resource, and $B$ denotes the total number of beds in the ER. The duration of the diagnostic procedure is then multiplied by this busyness factor to account for congestion.

**4. Event Scheduling and Time Advancement**

The model processes events in chronological order. An event is scheduled whenever a patient starts or finishes a task (arrives, completes triage, completes testing, etc.). The simulation clock then jumps to the time of the earliest scheduled event. This approach ensures the model efficiently captures transitions (e.g., “arrival,” “task completed,” “bed freed,” “shift changed”) without expending computational effort on idle time periods.

**5. Shift Changes and Reallocation**

During each day, shifts change every 8 hours. When a shift change occurs, the new number of on-duty physicians is reassessed. If the incoming shift has fewer physicians, patients currently assigned to departing physicians are assigned to the new physicians in a way that balances each physician’s workload.

**6. Initialization and Termination Criteria**

A warm-up period of 10 hours and a cool-down period of 3 hours are applied to the simulation. To reduce the impact of transient behavior during the warm-up and cool-down phases, data from the first and last day of the simulation are excluded from the KPI calculations. The KPIs are derived from a dataset spanning 45 days of simulated data.

**Appendix D: Variants of the Area Under the Curve Metric**

This appendix defines and compares several variants of the area under the curve (AUC) metric used to evaluate patient length-of-stay (LOS) performance across prioritization strategies. Each variant quantifies the cumulative percentage of patients meeting LOS thresholds over a specified time window but differs in how it weights early versus late portions of the distribution. By examining AUC variants with uniform, lower-threshold, and upper-threshold emphasis, analysts can capture distinct aspects of strategy performance—such as timely discharges for critical early patients or long-run clearance rates for overall throughput. All variants are normalized to the unit interval to ensure comparability across strategies and scenarios. A secondary purpose of this multi-definition framework is to conduct a sensitivity analysis: if the choice of AUC variant does not materially affect conclusions, then downstream comparisons are robust to how performance is aggregated across time thresholds.

**D.1 AUC**

In this study, we report four distinct definitions of the area under the LOS–threshold performance curve to emphasize different regions of the curve. Let $T_{\mathrm{mi}n}$ and $T_{\max}$​ denote the minimum and maximum threshold values (in minutes), and let $T_{P}(t)\in[0, 1]$ be the percentage of patients with LOS below $t$. We set $T_{\mathrm{mi}n}=5$ and $T_{\max}=12 \times60+5 = 725$ in the 12-hour version, and we set $T_{\max}=6 \times60+5 = 365$ in the 6-hour version.

Define the full‐range area as

$$AUC_{full}=\frac{1}{T_{\max}- T_{\mathrm{mi}n}}\int_{T_{\mathrm{mi}n}}^{T_{\max}} T_{P}(t) dt,$$

so that $AUC_{full}\in[0, 1]$.

**D.2 Weighted AUC with Emphasis on Lower Thresholds**
We introduce a weight function

$$w_{\mathrm{low}}\left( t \right)=\frac{T_{\max}-t}{\int_{T_{\mathrm{mi}n}}^{T_{\max}} {(T}_{\max}-u) du}=\frac{T_{\max}-t}{\frac{1}{2}\left( T_{\max}-T_{\mathrm{mi}n} \right)^{2}},$$

which integrates to one over ​$[T_{\mathrm{mi}n}, T_{\max}]$ and places greater mass near $T_{\mathrm{mi}n}$​. The corresponding weighted AUC is

$$AUC_{\mathrm{Low}}=\int_{T_{\mathrm{mi}n}}^{T_{\max}} w_{\mathrm{low}}\left( t \right) T_{P}(t) dt\in[0, 100].$$

**D.3 Weighted AUC with Emphasis on Higher Thresholds**
Analogously, define

$$w_{\mathrm{high}}\left( t \right)=\frac{t-T_{\min}}{\int_{T_{\mathrm{mi}n}}^{T_{\max}} (u-T_{\min}) du}=\frac{t-T_{\min}}{\frac{1}{2}\left( T_{\max}-T_{\mathrm{mi}n} \right)^{2}},$$

which biases mass toward $T_{\max}$​. Then

$$AUC_{\mathrm{High}}=\int_{T_{\mathrm{mi}n}}^{T_{\max}} w_{\mathrm{high}}\left( t \right) T_{P}(t) dt\in[0, 1].$$

Each variant preserves comparability across strategies by constraining its range to $[0, 1]$, but emphasizes different portions of the LOS‐threshold curve to capture early‐, mid-, or late-threshold performance characteristics.

**Appendix E: Illustrative Door-to-Doctor Time Examples**

This appendix provides a brief demonstration of how the evaluation framework can be applied to key performance indicators beyond LOS. Although LOS is used throughout the main manuscript for clarity, the framework is KPI-agnostic and can be used with any time-based service metric. We include three Door-to-Doctor Time (DTDT) examples to illustrate how the same techniques used in Section 3 readily extend to other indicators. These examples are intended only as a minimal demonstration of portability; they are not meant to represent a full DTDT analysis or to support any prescriptive conclusions.

Figure E.1 presents DTDT threshold-attainment curves aggregated across all patients. The interpretation mirrors that of the LOS curves in the main text: each line represents the proportion of patients whose DTDT falls below threshold $t$. As in the LOS setting, early- and late-threshold behavior differ meaningfully across strategies. Acuity-Based FCFS performs best at short horizons ($t<120$ minutes) but becomes less favorable at larger thresholds, whereas FCFS begins as one of the lowest-performing strategies yet eventually overtakes others as $t$ increases. These patterns demonstrate how threshold selection alone can shift apparent strategy rankings, reinforcing the need for standardized reporting across the full curve.

Figure E.2 shows an example of the elliptical utility visualization at a one-hour DTDT threshold. Points represent the percentage of low- and mid-acuity patients meeting the one-hour goal under each strategy, and the curves show level sets of the illustrative elliptical utility function $U_{1}=c$. The underlying hyperparameters ($\lambda=1.5$ for mid-acuity weighting; $\delta=0.3$ for overshoot penalization) are placeholders chosen solely for demonstration. Consistent with the threshold curves, Acuity-Based FCFS achieves the highest low-acuity DTDT at this threshold while maintaining comparable mid-acuity performance. No operational recommendations should be inferred from this figure; its role is to illustrate how stakeholder-defined utilities can be overlaid on any KPI.

Table E.1 reports four AUC variants—standard (0–12 h), half-range (0–6 h), early-emphasis, and late-emphasis—for DTDT threshold curves in the low- and mid-acuity cohorts. The relative ordering across AUC variants remains broadly stable. FCFS appears more favorable for low-acuity DTDT, whereas AAPQ and AAPQ-LWP perform best for mid-acuity DTDT. These results parallel the illustrative LOS findings and again highlight that the framework generalizes readily to alternative KPIs without modification.

Together, these examples demonstrate that the proposed evaluation framework is KPI-independent. Any time-based or target-based service measure—such as DTDT, LWBS, throughput times, or service-level benchmarks—can be incorporated using the same threshold, tail-risk, and utility analyses presented in the main manuscript.


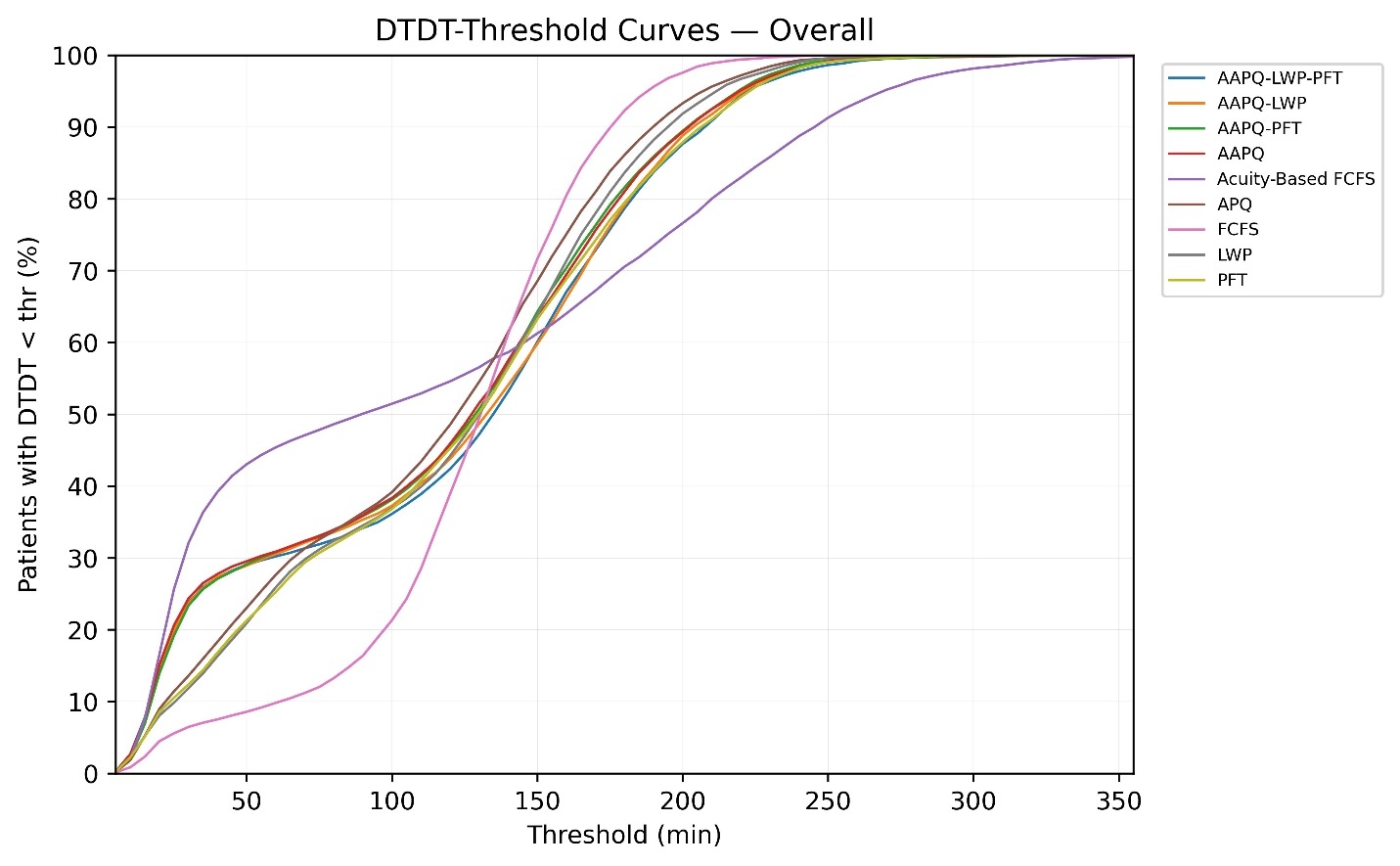


Fig. E.1 **Cumulative Discharge Profiles for All Patients.** This figure plots $\hat{T}_{DTDT}\left( t \right)$, the percentage of low-acuity patients discharged within $t$ minutes, for each prioritization strategy over a 6-hour window. The early-time advantage of Acuity-Based FCFS is evident for $t<120$ min, while its performance declines relative to other rules at longer thresholds. On the other hand, FCFS underperforms at low thresholds, but then becomes superior at higher thresholds.


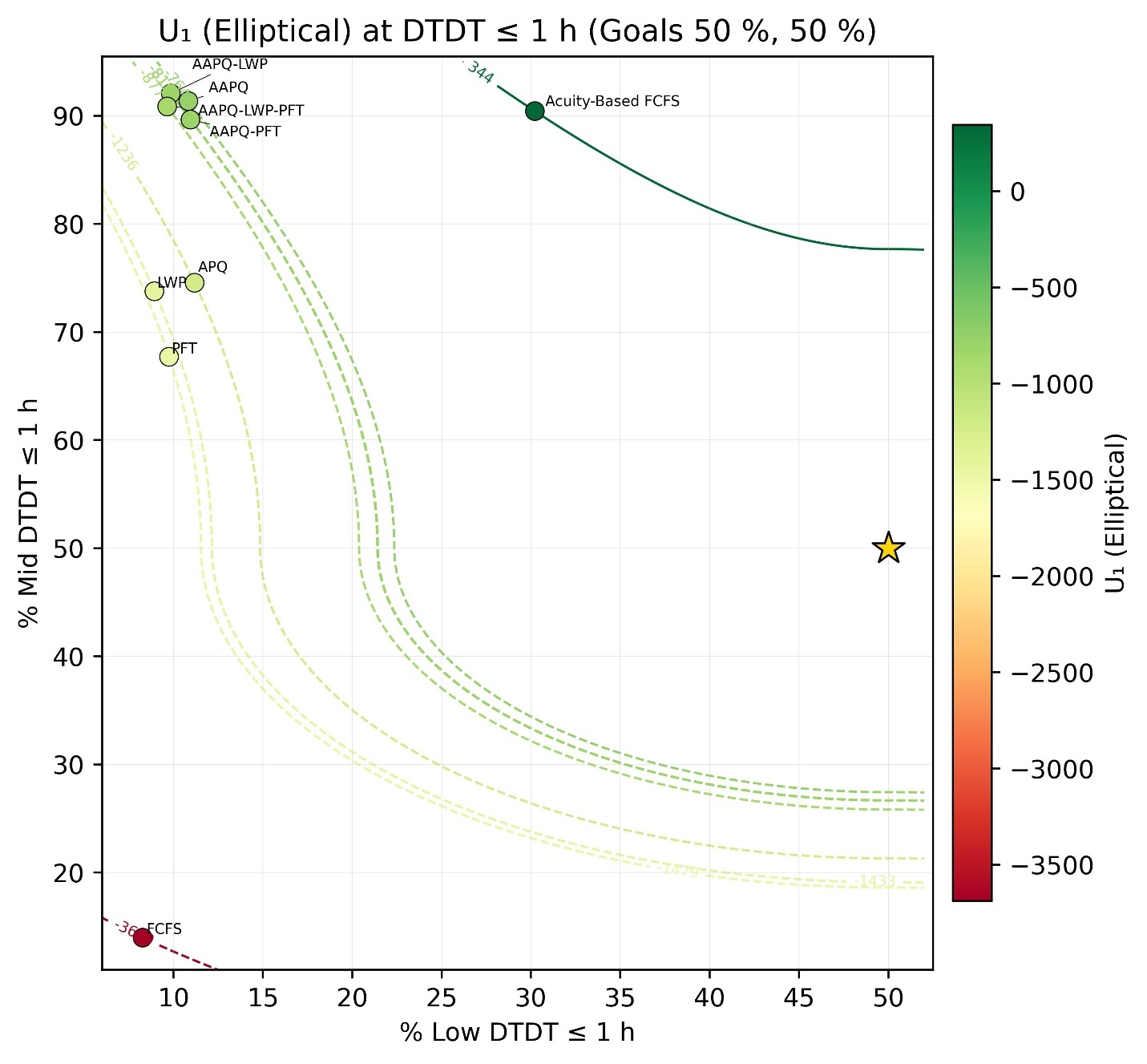


Fig. E.2 **Elliptical Utility Contours at** $\boldsymbol{t}^{\boldsymbol{*}}\boldsymbol{=1}$ **hour for Door-to-Doctor Time (DTDT).** The mid-acuity weighting hyperparameter is λ=1.5, the overshooting value is δ=0.3, and LOS goals are 100% of low-acuity patients and 90% of mid-acuity patients. Scatter plot of $\left( T_{DTDT-Low}\left( 1h \right),T_{DTDT-Mid}\left( 1h \right) \right)$ for each strategy, overlaid with elliptical-utility contours $U_{1}=c$. At this threshold, Acuity-Based FCFS maximizes low-acuity DTDT, with almost the same mid-acuity DTDT. Utility parameters are placeholders used solely to demonstrate visualization and interpretation; no prescriptive conclusion should be drawn from these settings without stakeholder elicitation.

Table E.1 **Area-Under-Curve (AUC) Metrics for Door-to-Doctor-Time (DTDT) Threshold-Based Discharge Curves**. This table reports four AUC variants—standard (0–12 h), half-range (0–6 h), early-emphasis (linearly decreasing weights), and late-emphasis (linearly increasing weights)—for low- and mid-acuity cohorts under each strategy. Despite different weighting schemes, rankings remain largely consistent; FCFS appears more favorable for low acuity patients whereas AAPQ and AAPQ-LWP do best for mid-acuity patients.

| **Group** | **Strategy** | **AUC (12h)** | **AUC (6h)** | **AUC (low emphasis)** | **AUC (high emphasis)** |
| --- | --- | --- | --- | --- | --- |
| Low | FCFS | 82% | 65% | 69% | 97% |
| Low | APQ | 81% | 63% | 67% | 96% |
| Low | Acuity-Based FCFS | 80% | 61% | 67% | 95% |
| Low | AAPQ-PFT | 80% | 61% | 66% | 96% |
| Low | LWP | 80% | 61% | 65% | 96% |
| Low | AAPQ | 80% | 61% | 66% | 96% |
| Low | PFT | 80% | 60% | 65% | 96% |
| Low | AAPQ-LWP | 80% | 59% | 65% | 96% |
| Low | AAPQ-LWP-PFT | 79% | 59% | 64% | 95% |
| Mid | AAPQ | 96% | 92% | 93% | 100% |
| Mid | AAPQ-LWP | 96% | 92% | 93% | 100% |
| Mid | AAPQ-LWP-PFT | 96% | 91% | 92% | 100% |
| Mid | Acuity-Based FCFS | 96% | 91% | 92% | 100% |
| Mid | AAPQ-PFT | 95% | 91% | 92% | 100% |
| Mid | APQ | 93% | 86% | 88% | 99% |
| Mid | LWP | 93% | 86% | 88% | 99% |
| Mid | PFT | 93% | 85% | 87% | 99% |
| Mid | FCFS | 83% | 67% | 71% | 97% |

**References**

1. Abdellaoui, M., H. Bleichrodt, and C. Paraschiv, *Loss aversion under prospect theory: A parameter-free measurement.* Management science, 2007. **53**(10): p. 1659-1674.

2. Kőszegi, B. and M. Rabin, *A model of reference-dependent preferences.* The Quarterly Journal of Economics, 2006. **121**(4): p. 1133-1165.

3. Walker, K., et al., *Has the implementation of time-based-targets for emergency department length-of-stay influenced the quality of care for patients? A systematic review of qualitative literature.* medRxiv, 2021: p. 2021.01. 03.21249171.

4. Tamiz, M., D. Jones, and C. Romero, *Goal programming for decision making: An overview of the current state-of-the-art.* European Journal of operational research, 1998. **111**(3): p. 569-581.

5. Jones, D. and M. Tamiz, *Practical goal programming*. Vol. 141. 2010: Springer.

6. Abohamad, W., A. Ramy, and A. Arisha. *A hybrid process-mining approach for simulation modeling*. in *2017 winter simulation conference (WSC)*. 2017. IEEE.

7. Anatolyev, S., *Dynamic modeling under linear-exponential loss.* Economic Modelling, 2009. **26**(1): p. 82-89.

8. Azimaee, P., M. Jafari Jozani, and Y. Maddahi, *Calibration of surgical tools using multilevel modeling with LINEX loss function: Theory and experiment.* Statistical Methods in Medical Research, 2021. **30**(6): p. 1523-1537.

9. Flavell, R., *A new goal programming formulation.* Omega, 1976. **4**(6): p. 731-732.

10. Wang, C.-N., N.-L. Nhieu, and T.T.T. Tran, *Stochastic chebyshev goal programming mixed integer linear model for sustainable global production planning.* Mathematics, 2021. **9**(5): p. 483.

11. Honari, H., M.G. Tamizi, and H. Najjaran. *Safety optimized reinforcement learning via multi-objective policy optimization*. in *2024 IEEE International Conference on Robotics and Automation (ICRA)*. 2024. IEEE.

12. Roy, A.D., *Safety first and the holding of assets.* Econometrica: Journal of the econometric society, 1952: p. 431-449.

13. Fishburn, P.C., *Mean-risk analysis with risk associated with below-target returns.* The American Economic Review, 1977. **67**(2): p. 116-126.

14. Rockafellar, R.T. and S. Uryasev, *Optimization of conditional value-at-risk.* Journal of risk, 2000. **2**: p. 21-42.

15. Stenson, B.A., J.S. Anderson, and S.R. Davis, *Staffing and provider productivity in the emergency department.* Emergency Medicine Clinics, 2020. **38**(3): p. 589-605.

16. Furian, N., et al., *Machine learning-based patient selection in an emergency department.* arXiv preprint arXiv:2206.03752, 2022.

17. Chaou, C.-H., et al., *Predicting length of stay among patients discharged from the emergency department—using an accelerated failure time model.* PloS one, 2017. **12**(1): p. e0165756.

18. Lee, S. and Y.H. Lee. *Improving emergency department efficiency by patient scheduling using deep reinforcement learning*. in *Healthcare*. 2020. MDPI.

19. Diefenbach, M. and E. Kozan, *Effects of bed configurations at a hospital emergency department.* Journal of Simulation, 2011. **5**(1): p. 44-57.
